# Variations in serum low-density lipoprotein and sST2 among heart failure patients with different ejection fraction groups and their clinical significance

**DOI:** 10.1097/MD.0000000000037357

**Published:** 2024-03-01

**Authors:** Yuanzhi Liu, Lijian Gao, Guangxian Zhao, Wenchen Zhang, Chuan Du, Wenjing Sun, Lei Jin, Hongyu Lu, He Zhou

**Affiliations:** a Department of Cardiology, Yanbian University Hospital, Yanji, China; b Coronary Artery Disease Center, Department of Cardiology, Fuwai Hospital, CAMS and PUMC, Beijing, China.

**Keywords:** ischemic cardiomyopathy, low-density lipoprotein, soluble ST2 protein

## Abstract

**Objective:**

This study aimed to examine the changes in serum Low Density Lipoprotein Cholesterol (LDL-C) and Soluble Growth Stimulating Expressed Gene 2 Protein (sST2) among Heart Failure (HF) patients with varying ejection fractions and their clinical significance, providing a reference for the clinical assessment of HF severity.

**Methods:**

A total of 238 HF patients treated in our hospital’s cardiology department from September 2019 to December 2021 were selected; 68 patients hospitalized in the same period were selected as the control group. General information, LDL-C and echocardiographic results of admitted patients were collected. According to LVEF results and the latest European Society of Cardiology standards in 2021, HF patients were categorized into those with HFpEF (n = 95), HFmrEF (n = 60), and HFrEF (n = 83). Meanwhile, venous blood was collected to determine sST2 and NT-proBNP to compare and analyze the changes and clinical significance of sST2 and LDL-C across the groups.

**Results:**

Compared to the control group, the HF group showed significant differences in age, gender, heart rate, smoking history, history of atrial fibrillation, history of diabetes, LVEDD, LVEF, sST2, and NT-proBNP levels (*P* < .05), but not in LDL-C levels. Significant differences (*P* < .05) were also found among the 3 HF groups in terms of age, gender, history of atrial fibrillation, LVEDD, LVEF, LDL-C, sST2, and NT-proBNP levels, with an increase in LVEDD, LDL-C, sST2, and NT-proBNP values as the ejection fraction decreased. ROC curve analysis indicated that the area under the curve (AUC) for sST2 in diagnosing HF was 0.915 (*P* < .05), with an optimal cutoff value of 23.71 ng/mL, a sensitivity of 76.5%, and a specificity of 95.6%; LDL-C was not a significant diagnostic marker for HF (*P* > .05). Coronary artery disease, NT-proBNP, and sST2 were identified as risk factors for HF. With each unit increase in coronary artery disease, the risk of HF increased by 36.3%; for NT-proBNP, the risk increased by 1.3% per unit; and for sST2, it increased by 18.3% per unit.

**Conclusion:**

As the ejection fraction decreases in HF patients, serum sST2 and LDL-C values progressively increase, which is clinically significant for predicting the severity of HF. sST2 is an independent risk factor for HF and can enhance the diagnostic accuracy for HF.

## 1. Introduction

Heart Failure (HF) represents the end-stage of coronary heart disease and has become one of the leading causes of mortality among these patients. Currently, cardiovascular diseases have the highest mortality rate, surpassing malignancies and other diseases.^[1]^ Globally, over 26 million individuals are afflicted with HF, with an incidence rate of about 0.2% per year in Western countries^[2]^; in China, the adult incidence rate is 1.3%, totaling approximately 8.9 million individuals.

Research indicates that the Soluble Growth Stimulating Expressed Gene 2 Protein (sST2) serves as a novel biomarker reflecting inflammation, fibrosis, and cardiac load and strain, holding significant relevance for the diagnosis and severity assessment of HF.^[3]^ Present studies on sST2 primarily utilize the widely applied NYHA cardiac function classification, which is subjectively biased and thus limited. Other studies have demonstrated a correlation between lipid levels and HF, suggesting that, like sST2, they could indicate the severity of HF. This study analyzed data from 238 HF patients treated in our hospital, with the results detailed below.

## 2. Subjects and methods

### 2.1. Study subjects

A total of 238 HF patients treated at Yanbian Hospital’s cardiology department from September 2019 to December 2021 were selected. General information, biochemical tests, and echocardiograms were collected. According to the “2021 European Society of Cardiology (ESC) Guidelines for the Diagnosis and Treatment of Acute and Chronic Heart Failure,” patients were classified into HFpEF (n = 95), HFmrEF (n = 60), and HFrEF (n = 83) groups. A control group of 68 individuals without coronary stenosis on Coronary Angiography, normal NT-proBNP values, and no abnormalities in other tests was also selected. The protocol was approved by the Ethics Committee (Signed by Chairman of the Ethics Committee Zhehu Jin).

#### 2.1.1. Inclusion criteria.

HF diagnosis criteria were based on the 2018 Chinese Heart Failure Guidelines and the 9th edition of “Internal Medicine” published by People’s Medical Publishing House, and all selected patients met the Framingham diagnostic criteria.^[4]^ The control group included patients who underwent Coronary Angiography in the cardiology department for health checkups during the same period, selected based on the absence of coronary stenosis and preoperative normal NT-proBNP values, along with normal results in other tests.

#### 2.1.2. Exclusion criteria.

Patients with acute or chronic severe infections; those with severe liver or kidney impairment due to various causes; cancer and other immunological disease patients; individuals with congenital heart disease, valvular disease, and cardiomyopathy; those with concurrent circulatory system diseases; patients with severe hematologic diseases; and non-consenting participants or those with incomplete clinical data collection.

### 2.2. Research methods

#### 2.2.1. Collection of general clinical data.

The general clinical data of the enrolled patients were collected and documented, including age, gender, heart rate, systolic and diastolic blood pressures, history of atrial fibrillation, smoking, coronary artery disease, hypertension, and diabetes.

#### 2.2.2. Specimen collection.

On the morning of the second day of admission, 2 to 3 mL of fasting venous blood from the elbow was drawn into EDTA anticoagulant tubes. Low-density lipoprotein cholesterol (LDL-C) biochemical indicators were measured using a German Roche Cobas 8000 fully automatic biochemical analyzer. Additionally, 4 to 5 mL was placed in a coagulant tube for sST2 testing. After coagulant tube samples had settled, a centrifuge (3000 rpm/15 minutes) was used to separate 300 µL of the upper serum into an EP tube and stored at −80°C. All samples were tested collectively post-collection to prevent freeze-thaw cycles.

#### 2.2.3. Echocardiography.

All patients underwent echocardiography using a color Doppler ultrasound within 48 hours of hospital admission. The entire procedure was conducted by professional physicians from our hospital’s ultrasound department, measuring the left ventricular end-diastolic diameter (LVEDD) and left ventricular ejection fraction (LVEF) and accurately recording the results.

#### 2.2.4. Serum sST2 measurement.

The sST2 assay was performed using a chemiluminescent immunological sandwich assay combined with magnetic particle separation. The operation was conducted using the Leadman CI2000 fully automatic chemiluminescent immunoassay analyzer, with the entire thawing and testing process managed by professional engineers from Beijing Leadman. All samples were calibrated before testing to ensure reliable and trustworthy results.

#### 2.2.5. Serum NT-proBNP measurement.

This method also employed a chemiluminescent immunological sandwich assay combined with magnetic particle separation. The procedure was carried out using a fluorescence quantitative immunoassay analyzer (Getein1600), operated throughout by experienced nurses from our department, ensuring reliable and credible outcomes.

### 2.3. Statistical methods

Data analysis was conducted using SPSS 26.0 software (Armonk, NY). For normally distributed data, group comparisons were made using *t* tests and one-way ANOVA, with trend tests using linear regression. For skewed data, the Mann–Whitney *U* test was used, with trend tests employing the Jonckheere–Terpstra test. Count data were analyzed using the χ^2^ test, with trend χ^2^ tests for trend analysis. Correlation employed Pearson’s or Spearman’s correlation coefficients. Logistic regression analysis was applied to factors influencing heart failure, and diagnostic value was assessed using ROC curves. A *P* value < .05 was considered statistically significant.

## 3. Results

### 3.1. Comparison of clinical data between groups

A comparison between the control group and HF groups revealed no significant statistical differences in history of hypertension, systolic pressure, diastolic pressure, and LDL-C levels (*P* > .05). However, significant differences were observed in the history of diabetes, smoking, atrial fibrillation, age, gender, heart rate, LVEDD, LVEF, sST2, and NT-proBNP (*P* < .05), as shown in Table 1.

**Table 1:** Comparison of general data between normal control group and HF group.

	CG (n = 68)	HF (n = 238)	*t*/χ^2^/Z	*P*
Age (yr)	62.07 ± 9.14	69.67 ± 12.00	5.611	<.001
Gender (female %)	44 (64.7)	91 (38.2)	15.032	<.001
Smoking history (%)	15 (22.1)	93 (39.1)	6.706	.010
History of hypertension (%)	38 (55.9)	148 (62.2)	0.881	.348
History of atrial fibrillation (%)	4 (5.9)	80 (33.6)	20.423	<.001
Diabetes history (%)	8 (11.8)	64 (26.9)	6.725	.010
Heart rate (beats/min)	80.85 ± 11.49	85.95 ± 17.50	2.837	.003
Systolic blood pressure (mm Hg)	132.88 ± 16.36	129.54 ± 22.25	1.364	.087
Diastolic blood pressure (mm Hg)	80.29 ± 10.75	80.07 ± 14.17	0.142	.444
LDL-C (mmol/L)	2.63 ± 0.89	2.46 ± 0.91	1.318	.094
LVEDD (mm)	44.46 ± 4.92	54.30 ± 9.70	11.363	<.001
LVEF (%)	61.87 ± 2.47	46.25 ± 12.05	10.617	<.001
sST2 (ng/mL)	13.59 (10.49–17.81)	47.47 (25.22–106.81)	10.440	<.001
NT-proBNP (pg/mL)	50.0 (50.0–50.0)	2848.5 (1456.0–7028.3)	12.082	<.001

CG = control group, HF = heart failure.

### 3.2. Comparison of clinical data among heart failure groups with different ejection fractions

The comparison of clinical data among HF groups with different ejection fractions showed statistical significance in age, gender, history of atrial fibrillation, LVEDD, LVEF, LDL-C, sST2, and NT-proBNP values (*P* < .05). With decreasing ejection fractions, levels of LVEDD, LDL-C, sST2, and NT-proBNP progressively increased; there were no statistical differences among the 3 groups concerning heart rate, history of hypertension, systolic pressure, diastolic pressure, smoking history, history of coronary heart disease, and diabetes (*P* > .05), as indicated in Table 2.

**Table 2:** HF clinical data comparison among HF patients with different ejection fractions.

	HFpEF (n = 95)	HFmrEF (n = 60)	HFrEF (n = 83)	*P*1	*P* trend
Age (yr)	72.49 ± 9.64	68.10 ± 13.14	67.57 ± 13.03	.011	.006
Gender (female %)	46 (48.4)	19 (31.7)	26 (31.3)	.031	.017
Smoking history (%)	31 (32.6)	27 (45.0)	35 (42.2)	.237	.182
History of hypertension (%)	64 (67.4)	37 (61.7)	47 (56.6)	.336	.141
History of atrial fibrillation (%)	40 (42.1)	20 (33.3)	20 (24.1)	.040	.011
History of coronary heart disease (%)	66 (69.5)	43 (71.7)	63 (75.9)	.629	.342
Diabetes history (%)	21 (22.1)	18 (30.0)	25 (30.1)	.398	.222
Heart rate (beats/min)	87.02 ± 21.41	88.17 ± 15.93	83.12 ± 12.80	.175	.138
SBP (mm Hg)	130.72 ± 20.99	133.33 ± 24.25	125.45 ± 21.75	.089	.114
DBP (mm Hg)	79.27 ± 12.80	81.52 ± 12.25	79.93 ± 15.62	.629	.759
LDL-C (mmol/L)	2.31 ± 0.85	2.49 ± 0.76	2.65 ± 1.04	.045	.021
LVEDD (mm)	46.68 ± 5.81	55.90 ± 8.31	61.87 ± 7.52	<.001	<.001
LVEF (%)	58.73 ± 4.38	44.85 ± 2.77	32.98 ± 5.64	<.001	<.001
sST2 (ng/mL)	36.23 (21.31–87.18)	48.44 (23.13–127.04)	54.91 (31.28–148.34)	.014	.003
NT-proBNP (pg/mL)	2147.0 (953.0–5222.0)	2332.0 (1481.3–5788.3)	4599.0 (2258.0–12962.0)	<.001	<.001

HFmrEF = Heart Railure with Mildly Reduced Ejection Fraction, HFpEF = Heart Failure with Preserved Ejection Fraction, HFrEF = Heart Failure with Reduced Ejection Fraction.

### 3.3. Diagnostic value of serum sST2 and LDL-C for heart failure

**Figure 1.**
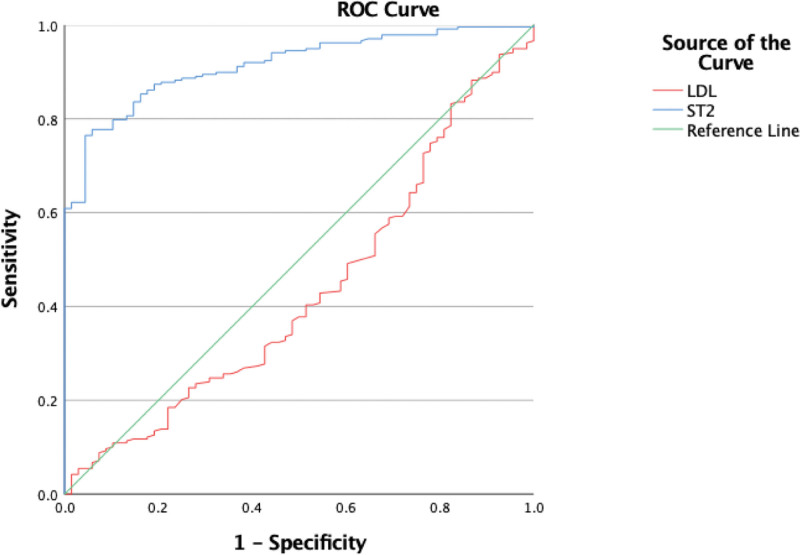
ROC curve analysis of the diagnostic value of sST2 and LDL-C in heart failure. The diagnostic value of heart failure was analyzed by ROC curve, in which the green line represented the reference line, the red curve represented the value of LDL-C (mmol-L), the blue curve represented the value of sST2 (ng/mL), and the horizontal axis represented the specificity and the vertical axis represented the sensitivity type. It was generally considered that *P* < .05 was statistically significant. The area under ROC curve for sST2 diagnosis of HF was 0.915 (*P* < .05), the optimal critical value was 23.71 ng/mL, the sensitivity was 76.5%, and the specificity was 95.6%. LDL-C had no significance in the diagnosis of HF (*P* > .05). HF = Heart Failure, LDL-C = low density lipoprotein cholesterol, sST2 = Soluble Growth Stimulating Expressed Gene 2 Protein.

ROC curve analysis demonstrated that the area under the ROC curve for sST2 in diagnosing HF was 0.915 (*P* < .05), with an optimal cutoff value of 23.71 ng/mL, a sensitivity of 76.5%, and a specificity of 95.6%. LDL-C was not significant for the diagnosis of HF (*P* > .05), as presented in Figure 1.

### 3.4. Logistic regression analysis of factors influencing heart failure

The logistic regression analysis indicated that coronary heart disease, NT-proBNP, and sST2 are risk factors for HF. With each unit increase in coronary heart disease, the risk of heart failure increased by 36.3%; each unit increase in NT-proBNP was associated with a 1.3% increased risk; and each unit increase in sST2 was associated with an 18.3% increased risk, as seen in Table 3.

**Table 3:** Logistics regression analysis of factors affecting heart failure.

	B	SE	Wald	*P*	OR	95% CI
Age	−0.008	0.057	0.022	.883	0.992	0.887–1.109
History of hypertension	−0.080	1.034	0.006	.939	0.924	0.122–7.004
History of atrial fibrillation	−0.153	1.448	0.011	.916	0.858	0.050–14.666
History of coronary heart disease	3.345	1.131	8.748	.003	28.363	3.091–260.270
Heart rate	−0.010	0.031	0.104	.748	0.990	0.933–1.051
LDL-C	0.168	0.476	0.124	.725	1.183	0.465–3.009
LVEF	−0.278	0.207	1.794	.180	0.757	0.504–1.137
NT-proBNP	0.013	0.005	8.816	.003	1.013	1.005–1.022
sST2	0.168	0.067	6.374	.012	1.183	1.038–1.348

## 4. Discussion

Heart failure (HF) is a severe clinical syndrome within cardiovascular diseases and a leading cause of mortality in patients with coronary artery disease. Currently, B-type natriuretic peptide (BNP) and N-terminal-pro BNP (NT-proBNP) are the mainstays of HF diagnosis. However, their accuracy is somewhat limited due to variability with age, gender, and renal function. Increasing evidence suggests that soluble ST2 (sST2) holds greater value in the diagnosis and severity assessment of HF. The ST2 protein, a member of the interleukin-1 (IL-1) receptor superfamily known as growth stimulation expressed gene 2, was identified by Tominaga in 1989, with Schmitz confirming in 2005 that IL-33 is the endogenous ligand for ST2.^[5]^ There are 2 primary isoforms of ST2: the transmembrane or cellular form (ST2L) and the soluble or circulating form (sST2). The IL-33/ST2L signaling pathway plays a cardioprotective role, including delaying the onset of atherosclerosis, anti-myocardial fibrosis, reducing myocardial cell death, and improving prognosis. sST2 acts as a “decoy receptor” that, when released in response to mechanical stress, can inhibit the cardioprotective effects of the IL-33/ST2L signaling pathway.^[3,6]^ In recent years, due to extensive research on sST2, its potential in the diagnosis of heart failure (HF) has become increasingly promising. Huang et al^[7]^ discovered through a meta-analysis that sST2 could be significant in the diagnosis of HF. Aldous et al^[8]^ evaluated the diagnostic relevance of sST2 for acute heart failure and found that it had a diagnostic sensitivity of 73.5% and specificity of 79.6%, with sST2 demonstrating greater specificity for HF diagnosis than NT-proBNP (66.2%). The results of this study indicate that in comparison between the control group and the HF group, a decrease in LVEF and an increase in sST2 levels were observed. Furthermore, Receiver Operating Characteristic (ROC) curve analysis also highlighted the importance of sST2 in HF diagnosis.

sST2 and the severity of HF soluble ST2 (sST2) is also crucial for assessing the severity of heart failure (HF). Research indicates that the ST2/IL-33 signaling pathway is a mechanistically activated system. During the development of HF, decreased myocardial contractility and ejection fraction lead to an increase in the volume of blood remaining in the ventricles at the end of diastole, causing ventricular mechanical expansion, which, in turn, elevates the expression levels of sST2. This series of signaling events reduces the protective effect of ST2L on myocardial cells, culminating in myocardial fibrosis and ventricular remodeling. The American College of Cardiology and the American Heart Association, in 2013, accorded sST2 a IIb evidence level for risk stratification. Jin et al^[9]^ observed in a study of 176 HF patients – categorized by NYHA class into 57 class IV, 59 class III, and 60 class II cases – and 60 control subjects that sST2 levels were significantly higher in the HF group compared to controls and increased progressively with worsening cardiac function. This suggests that sST2 is a biomarker for the severity of HF. Wang et al^[10]^ found a correlation between sST2 levels, patient LVEF, and NYHA classification, noting that as sST2 levels rose, LVEF decreased, and NYHA classification increased. sST2 also emerged as an independent risk factor for HF, indicating a close relationship between sST2 levels and HF severity. Domestic studies have also revealed that elevated sST2 levels in HF patients are closely linked to the severity of the condition.^[11–15]^ A meta-analysis found that sST2 can assess the severity of HF and predict adverse outcomes and cardiovascular events in patients with chronic HF.^[16]^ In the present study, across 3 HF groups, an increasing trend in sST2 was observed with decreasing LVEF, which is primarily used clinically to evaluate patient ejection fraction – a decrease in which indicates reduced myocardial contractile function and heightened HF severity^[17]^, thus underscoring the relevance of sST2 levels in assessing the severity of a patient’s HF condition.

Lipids and the Severity of HF Elevated LDL-C levels can exacerbate the development of atherosclerosis and increase blood viscosity, thereby intensifying ischemic and hypoxic symptoms in HF patients and leading to further deterioration of cardiac function^[18–23]^. Studies have shown that LDL-C levels tend to rise with the increasing severity of HF. Pharmacological reduction of LDL-C can enhance cardiac function, alleviate symptoms, and improve treatment efficacy in patients with HF, suggesting a relationship between LDL-C levels and the severity of HF. Li Tingting et al^[24]^ found in a study of 70 patients with chronic heart failure at the Huaibei People’s Hospital that while lipid levels were not an independent risk factor for mortality in elderly patients with heart failure, they could reflect the severity of the disease. In our current study, no statistical difference was found when comparing LDL-C levels between the normal control group and the HF group. However, within the HF cohorts, LDL-C levels gradually increased as ejection fraction decreased. This indicates that LDL-C is not diagnostic for HF patients but can be used to assess the severity of the condition in those with a confirmed diagnosis.

## 5. Conclusion

In summary, within HF patients, both sST2 and LDL-C levels exhibit an increasing trend as ejection fraction decreases, serving as valuable indicators of the severity of heart failure; sST2 is an independent risk factor for HF and enhances the accuracy of its diagnosis. This study has certain limitations: it is confined to a single hospital with a relatively small sample size and lacks regular follow-up with discharged patients. Further research should involve multicenter, large-scale studies with regular follow-ups post-discharge to ascertain their prognostic significance. Additionally, some patients have a long-term history of treatment in cardiology with statins, which may affect their lipid levels and warrants further investigation for a comprehensive understanding.

## Author contributions

**Conceptualization:** Guangxian Zhao.

**Data curation:** Yuanzhi Liu, Wenchen Zhang, Wenjing Sun.

**Formal analysis:** Guangxian Zhao.

**Investigation:** Guangxian Zhao.

**Methodology:** Yuanzhi Liu, Guangxian Zhao.

**Project administration:** Yuanzhi Liu.

**Resources:** Yuanzhi Liu, Guangxian Zhao, Wenchen Zhang, Chuan Du, Wenjing Sun, Lei Jin, Hongyu Lu, He Zhou.

**Software:** Yuanzhi Liu, Guangxian Zhao.

**Supervision:** Lijian Gao, Guangxian Zhao.

**Validation:** Yuanzhi Liu, Guangxian Zhao.

**Visualization:** Yuanzhi Liu, Guangxian Zhao.

**Writing – original draft:** Yuanzhi Liu.

**Writing – review & editing:** Yuanzhi Liu, Guangxian Zhao.
